# *CERKL*, a Retinal Disease Gene, Encodes an mRNA-Binding Protein That Localizes in Compact and Untranslated mRNPs Associated with Microtubules

**DOI:** 10.1371/journal.pone.0087898

**Published:** 2014-02-03

**Authors:** Alihamze Fathinajafabadi, Eva Pérez-Jiménez, Marina Riera, Erwin Knecht, Roser Gonzàlez-Duarte

**Affiliations:** 1 Laboratorio de Biología Celular, Centro de Investigación Príncipe Felipe, Valencia, Spain; 2 Departament de Genètica, Facultat de Biologia, Universitat de Barcelona, Barcelona, Spain; 3 Centro de Investigación Biomédica en Red de Enfermedades Raras (CIBERER), Spain; National Eye Institute, United States of America

## Abstract

The function of *CERKL* (CERamide Kinase Like), a causative gene of retinitis pigmentosa and cone-rod dystrophy, still awaits characterization. To approach its cellular role we have investigated the subcellular localization and interaction partners of the full length CERKL isoform, CERKLa of 532 amino acids, in different cell lines, including a photoreceptor-derived cell line. We demonstrate that CERKLa is a main component of compact and untranslated mRNPs and that associates with other RNP complexes such as stress granules, P-bodies and polysomes. CERKLa is a protein that binds through its N-terminus to mRNAs and interacts with other mRNA-binding proteins like eIF3B, PABP, HSP70 and RPS3. Except for eIF3B, these interactions depend on the integrity of mRNAs but not of ribosomes. Interestingly, the C125W CERKLa pathological mutant does not interact with eIF3B and is absent from these complexes. Compact mRNPs containing CERKLa also associate with microtubules and are found in neurites of neural differentiated cells. These localizations had not been reported previously for any member of the retinal disorders gene family and should be considered when investigating the pathogenic mechanisms and therapeutical approaches in these diseases.

## Introduction

Retinitis pigmentosa (RP) is the most common form of retinal dystrophies (prevalence of 1∶4,000) and is characterized by night blindness and progressive loss of vision due to photoreceptor degeneration [1]. RP is a hereditary disorder with extremely high genetic heterogeneity and to date more than fifty causative genes have been identified. Among them, *CERKL* (CERamide Kinase Like) was first identified in a RP Spanish family [2] and later was shown to promote cone-rod dystrophy (CRD), a retinal disorder associated with a more severe phenotype [3]. *CERKL* encodes a protein particularly abundant in cones and also present in rods and at the inner nuclear layer and ganglion cell layer [4], [5]. CERKL function, in spite of many studies, is still unknown. Of the many isoforms reported in human and mouse retina [6], only CERKLa (hereafter called simply CERKL), the full length protein that encompasses all thirteen exonic regions and comprises 532 amino acids, is conserved in vertebrates [7].

CERKL belongs to the ceramide kinase (CERK) protein family, whose members share, in addition to a Pleckstrin homology (PH) domain, an evolutionary conserved diacylglycerol kinase domain presumed to be involved in sphingolipid metabolism [8]. Although the physiological function of this domain in CERKL has been deeply investigated *in vivo* and *in vitro* by different groups [8], [9], [10], [11], no evidences have been gathered for such a role in this protein [12]. Also, two nuclear localization signals and two nuclear export signals have been proposed to regulate a nucleus/cytoplasm traffic of CERKL [8], [9], [13], a process that needs to be accommodated in the function of this protein.

To gain further insights into the cellular role of CERKL we have investigated its subcellular localization and its interaction partners in different cell lines, including the photoreceptor-derived cell line 661W. CERKL was found to colocalize, in a nuclear import/export-dependent manner, with stress granules (SGs), which are aggregates that appear when the cells are under stress, composed of specific proteins (PABP, poly(A) binding protein, and several initiation factors such as eIF4E, eukaryotic translation initiation factor 4E, among others), small but not large ribosomal subunits and polyadenylated mRNAs [14]. CERKL was associated also with P-bodies (PBs), which are involved in mRNA turnover [15], to polysomes and mainly to compact untranslated messenger ribonucleoprotein particles (mRNPs). CERKL was found to directly interact through its N-terminal region with mRNA and also to bind to some proteins of the translation machinery. In addition, these compact mRNPs also bind to microtubules and are found in neural differentiated cells. Although there are other RP-causing genes that encode spliceosome components or splicing factors, *CERKL* becomes the first gene that encodes a protein that binds to mature mRNAs in the cytoplasm.

## Results

### CERKL localizes to SGs and P-bodies

**Figure 1:**
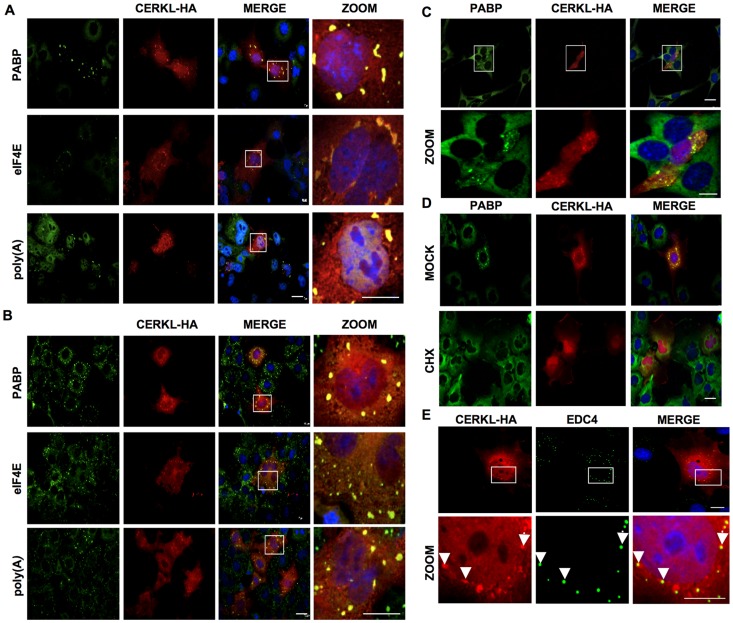
CERKL localizes to SGs and P-bodies. **A** and **B**) Colocalization of CERKL (HA) with three markers of SGs (PABP and eIF4E, detected with specific antibodies described in the Materials and Methods section), and the mRNAs poly(A) tail, detected with oligo(dT) FISH (upper, middle and lower panels, respectively) in COS-7 cells overexpressing CERKL-HA, either untreated (**A**) or incubated with 500 µM sodium arsenite for 30 min (**B**). **C**) The localizations of PABP and CERKL (HA) in transfected 661W mouse photoreceptor derived cells were compared by immunofluorescence. Images at higher magnification of the rectangles in the upper row are shown below. **D**) COS-7 cells transfected with CERKL-HA were untreated (MOCK) or treated for 30 min with 100 µg/mL cycloheximide (CHX) and the localizations by immunofluorescence of PABP and CERKL (HA) were compared. **E**) COS-7 cells were transfected with CERKL-HA and treated with sodium arsenite as in **B**. The localizations by immunofluorescence of CERKL (HA) and the P-body marker EDC4 were compared. Images at higher magnification of the rectangles in the upper row are shown below. Arrowheads indicate various colocalizations. All bars: 10 µm.

Upon transient transfection, CERKL was found in COS-7 cells both in the cytoplasm and in the nucleus. In the cytoplasm, although the major part of CERKL was uniformly distributed, it was also found in the form of aggregates (see Fig. 1A, HA panels). Therefore, we tried to identify these aggregates using a battery of organelle markers. These aggregates containing CERKL were partially found around the nuclear envelope and endoplasmic reticulum and they were absent from the Golgi complex (*cis* and *trans* Golgi), mitochondria, early, late and recycling endosomes, clathrin-coated vesicles, lysosomes and autophagosomes (Fig. S1). Also, these structures neither corresponded to lipid droplets, as there was no colocalization of CERKL with BODIPY 493 or with a well established lipid droplet protein (adipose differentiation-related protein, ADFP), nor to aggregates of misfolded proteins, as there was no colocalization of CERKL with p62 or with polyubiquitinated proteins (data not shown). However, CERKL clearly colocalized with markers of SGs such as poly(A) tails, PABP, eIF4E, RPS3 (ribosomal protein S3) and G3BP1 (Ras GTPase-activating protein-binding protein 1) (Fig. 1A and data not shown).

Although the granules containing CERKL were already observed in transiently transfected COS-7 cells, their number increased about 1.5 times under stronger stress conditions such as exposure to 500 µM sodium arsenite (SA)(Fig. 1B and Fig. S2 upper panel, 14.8±3.5 *vs* 9.7±3.2 per cell, n = 100). Under heat-shock (as shown for HeLa cells in Fig. S4B) colocalization of CERKL with SGs was also evident and coupled to a higher number of CERKL-containing SGs, respectively) (Fig. 1B). This localization was specific for CERKL, since overexpressed GFP did not colocalize under any condition with SGs (Fig. S3). In addition, the localization of CERKL to SGs could be also extended to other cell types, such as human HeLa cells (Fig. S4) and, of more relevance since it is in the photoreceptors where *CERKL* mutations exert their pathological effects [1], the mouse photoreceptor derived cell line 661W (Fig. 1C).

In the presence of cycloheximide, an inhibitor of protein synthesis that causes ribosome stalling, SGs are not further formed and become disassembled [14]. Therefore, the localization of CERKL to SGs was lost when this inhibitor was added to both untreated (Fig. 1D) and SA-treated (Fig. S2) transiently transfected COS-7 cells and under these conditions the protein was mainly found diffuse in the cytosol. Similar results were obtained with HeLa cells exposed to heat-shock (Fig. S4B, two lower panels). These changes were not due to variations in CERKL, as the total level of overexpressed CERKL under heat-shock stress was minimally affected by the cycloheximide treatment (Fig. S4C).

SGs are in dynamic equilibrium with PBs, which are cytoplasmic organelles involved in mRNA degradation [15]. Figure 1E shows an example of the colocalization of CERKL with EDC4, a marker of PBs, in transiently transfected COS-7 cells after stress induction with SA. The same colocalization was also observed in other cells (data not shown).

In summary, the results above show that CERKL, in addition to be found in the nuclei, has two major patterns of localization in the cytoplasm, diffuse in the cytosol and aggregated in SGs and PBs, a localization that resembles that of proteins involved in mRNA metabolism [16].

### CERKL targeting to SGs requires its nuclear import/export and mRNA transcription

**Figure 2:**
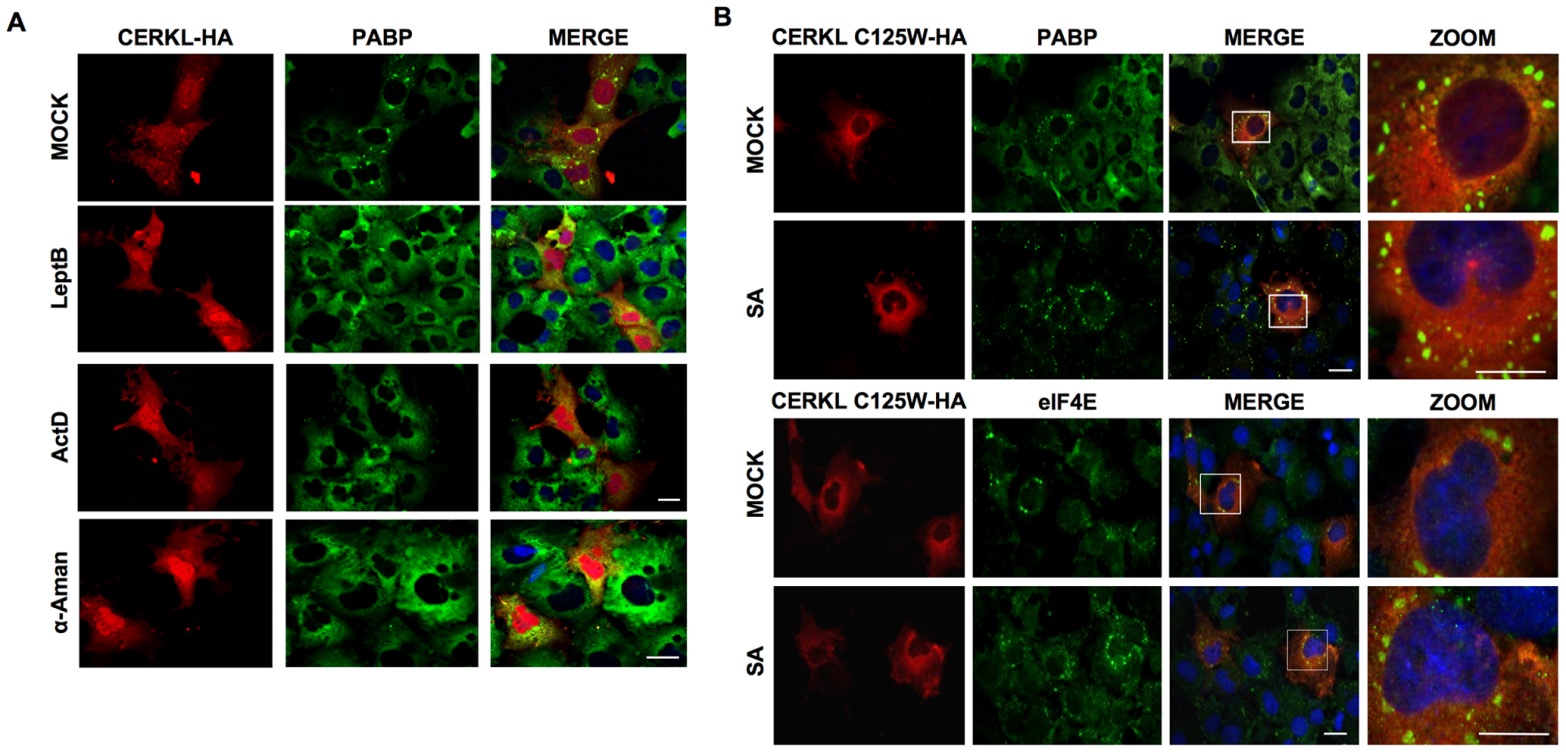
Formation of SGs by overexpression of CERKL requires the nuclear import/export of the protein. **A**) COS-7 cells overexpressing CERKL-HA were mock-treated (upper row) or treated for 2 h with 40 nM leptomycin B (LeptB), 1 µg/mL actinomycin D (ActD) or 100 µg/mL alpha-amanitin (α-Aman). Then, the localizations of CERKL (HA) and PABP (marker of SGs) were investigated by immunofluorescence. **B**) COS-7 cells overexpressing the CERKL-C125W mutant were mock-treated or treated with 500 µM SA for 30 min and the localizations of the mutant CERKL (CERKL C125W-HA) and PABP (upper panels) or eIF4E (lower panels) were investigated by immunofluorescence. Images at higher magnification of the rectangles are shown on the right. All bars: 10 µm.

Two nuclear localization signals and two nuclear export signals have been proposed to regulate CERKL trafficking between the cytoplasm and the nucleus [8], [9], [13]. The nuclear export of CERKL is mediated by binding of the nuclear export signal to CRM1, as evidenced by the accumulation of CERKL in the nucleus after treatment with leptomycin B (Fig. 2A and data not shown). In addition, this treatment confirmed that the localization of CERKL to SGs requires the export of the protein from the nucleus. Moreover, when the transcription of DNA was inhibited with actinomycin D, the CERKL aggregates disappeared and CERKL accumulated in the nuclei. The same result was obtained when RNA polymerase II was inhibited with α-amanitin (Fig. 2A), indicating that export of CERKL and its localization to SGs depends not only on the export of the protein from the nucleus but also on an active mRNA transcription.

We observed that a point mutant (C125W) within the PH domain of CERKL, which occurs in patients of CRD, was unable to enter the nucleus and remained in the cytoplasm (HA panels in Fig. 2B). Therefore, we took advantage of this mutant to confirm the requirement of the shuttling of CERKL between nucleus and cytoplasm for its localization to SGs. As shown in Figure 2B, the CERKL-C125W mutant did not show any association to SGs even after treating the cells with SA.

Therefore, all these results support a dynamic CERKL cycle of nuclear import/export, which depends on mRNA transcription and that is necessary for CERKL targeting to SGs. This cycle is absent in a pathogenic mutant (C125W) of CERKL.

### In the absence of stress, most CERKL associates with polysomes and compact and untranslated mRNP particles

**Figure 3:**
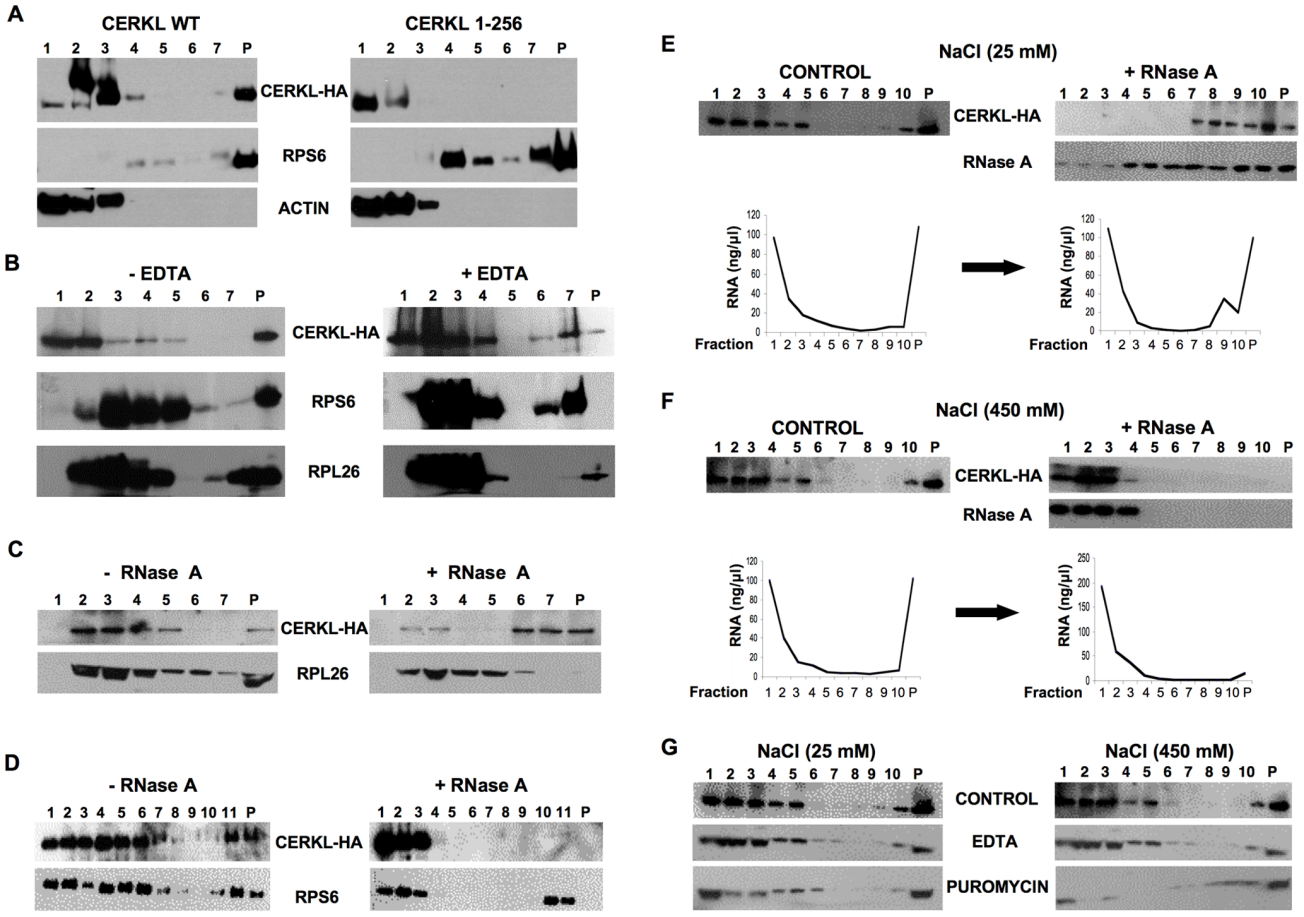
CERKL localizes to polysomes and compact mRNPs. **A**) HeLa cells stably expressing CERKL WT-HA or an N-terminal fragment of the protein (CERKL 1-256-HA), generated as described in the Materials and Methods section, were lysed and polysomes were isolated by centrifugation in a 0.5-1.3-1.7-2.1 M discontinuous sucrose gradient. Seven 1 ml fractions (1–7) and the pellet (P) were analyzed by immunoblot using antibodies that recognize HA, the ribosomal protein RPS6. Actin was used as control. **B** and **C**) Similar experiments were carried out incubating the cell lysates with EDTA (15 mM) (**B**) or RNase A (100 µg/mL) (**C**) before centrifugation. Antibodies that recognize HA and the ribosomal proteins RPS6 and RPL26 were used. **D**–**F**) The pellet (**D**) and fractions 2 and 3 of the gradient (**E** and **F**) were treated with 25 mM (**D** and **E**) or 450 mM (**F**) NaCl, with or without RNase A (100 µg/mL) and subsequently centrifuged in a continuous sucrose density gradient (0.3–1.5 M) as described in Materials and Methods. The new fractions (1–11, in **D** and 1–10, in **E** and **F**) and the pellet (P) were immunoblotted with HA and RPS6 (**D**) or RNase A (**E** and **F**) antibodies. Below the Western blots, the concentration of RNA quantified at 260 nm is shown. **G**) The same experiment as in **E** and **F**, with fractions 2 and 3 treated with 30 mM EDTA or 1 mM puromycin at 25 mM and 450 mM NaCl before the continuous sucrose gradient. Ten fractions and the pellet were analyzed by immunoblot using antibodies that recognize HA.

As mentioned before, SGs are in dynamic equilibrium with polysomes. Since CERKL colocalized with SGs but it was mainly found with a uniform distribution in the cytosol, we explored the possibility that this protein was also present in polysomes. In the absence of stress, HeLa cells that stably express CERKL-HA did not form SGs (Fig. S4A and B), in contrast to transiently transfected cells. Therefore, we isolated polysomes from these cells in a discontinuous sucrose gradient and analyzed the different fractions by Western blot. Figure 3A–C (left panels) shows that CERKL (amino acids 1–532, CERKL WT) in the cytosol localizes in the pellet (P) of polysomes (detected with antibodies that recognize proteins from the small or the large ribosomal subunits, RPS6 or RPL26, respectively). In contrast, a CERKL truncated protein (amino acids 1–256, CERKL 1–256) that does not shuttle between the nucleus and the cytoplasm [2] did not associate with the polysomal pellet (Fig. 3A, right panels). In addition to the pellet, CERKL WT was also found, and to a higher extent, in the fractions above it (Fig. 3A, upper left panel).

As expected, the colocalization of CERKL with polysomes was lost after treatment of the lysates with 15 mM EDTA (Fig. 3B), but surprisingly not after a treatment with ribonuclease A (RNase A, Fig. 3C). Moreover, in the latter condition a shift of CERKL from the lighter to the heavier fractions of the gradient was observed (see Fig. 3C, CERKL WT + RNase A). Therefore, we next treated separately with RNase A the polysomal pellet (P in Fig. 3A–C) and two of the fractions above containing most of the CERKL protein (fractions 2 and 3 in Fig. 3A–C) and subjected them to a second centrifugation in a continuous sucrose gradient. The association of CERKL to the original pellet (polysomes) was now sensitive, as expected, to the RNase A treatment and CERKL moved to the lighter sucrose fractions (Fig. 3D). However, CERKL in the two original soluble fractions 2 and 3 accumulated under our standard conditions (25 mM NaCl) in the heavier fractions and in the pellet of the continuous sucrose gradient (Fig. 3E). In these fractions, RNase A and RNA also accumulated (Fig. 3E, see Western blot below in the right panel and the line graphs below), because RNase A forms complexes [17] that can trap the mRNAs and their associated proteins. This explains the presence of CERKL in the heavier fractions and in the pellet from the lysates treated with RNase A (Fig. 3C).

We reasoned, therefore, that the soluble fractions contain CERKL and mRNA in a quite folded conformation that results in a compact and RNase A-insensitive structure. Accordingly, when RNase A was added to these same fractions in the presence of a high salt concentration (450 mM), CERKL, together with RNase A and RNA, were displaced to the lighter fractions of the continuous density sucrose gradient (Fig. 3F, compare with Fig. 3E). This result confirms the compact nature of these particles, which only become relaxed and RNase A-sensitive in the presence of a high salt concentration. In addition, treatment of these fractions with 30 mM EDTA or with 1 mM puromycin did not change the CERKL distribution in the gradient at any salt concentration (Fig. 3G). This indicates the absence of whole ribosomes and, therefore, that translation does not take place in these complexes.

### CERKL is an mRNA binding protein that interacts with components of the translation machinery

**Figure 4:**
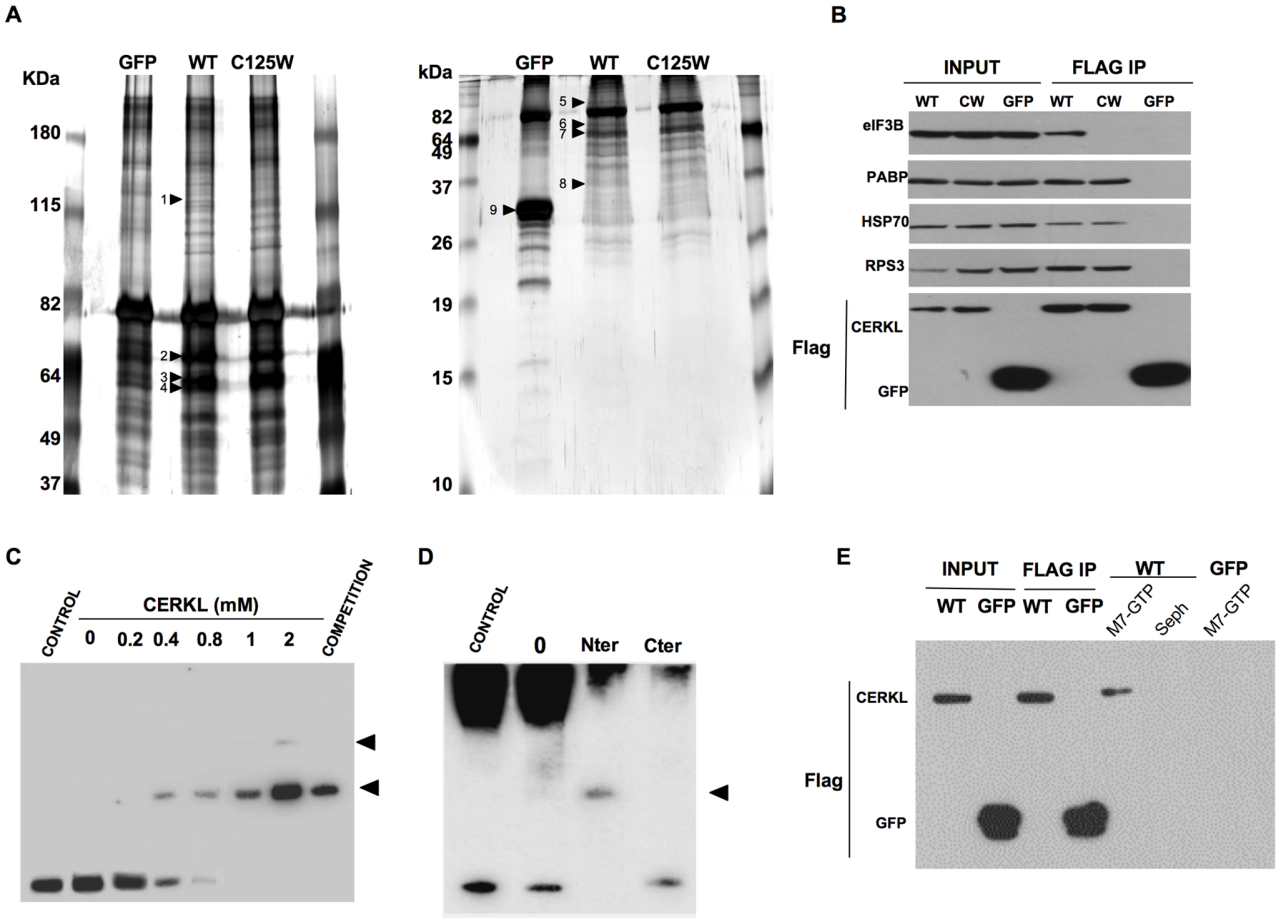
CERKL interacts with components of the translation machinery and with mRNAs. **A**) HEK-293T cells overexpressing Flag-tagged GFP, CERKL- WT or CERKL-C125W were lysed and Flag was immunoprecipitated as described in Materials and Methods, analyzed by SDS-PAGE in 8% (left panel) and 20% (right panel) polyacrylamide gels and silver-stained. Proteins were characterized by mass spectrometry (see Materials and Methods). The positions of the proteins are indicated by arrows, from up to down as follows: eIF3B (1), CERKL (2), alpha-tubulin (3), ß-tubulin (4) (8%polyacrylamide gel), PABP (5), HSP70 (6), CERKL (7) and RPS3 (8) (20% polyacrylamide gel, WT lane) and GFP (9) (20% polyacrylamide gel, GFP lane). **B**) Flag immunoprecipitations (FLAG IP) of protein homogenates from HEK 293T cells transfected with CERKL-Flag (WT), CERKL-C125W-Flag (CW) or, as a control, GFP-Flag (GFP). The position of CERKL and GFP, detected with anti-Flag, is shown. Specific antibodies were used to detect eIF3B, PABP, HSP70 and RPS3. **C**) EMSA of CERKL protein incubated at the indicated concentrations with biotinylated mRNAs from human retina. BSA (CONTROL) and His-MBP (0) were used as negative controls. Addition of an excess of non-biotinylated probes reduced the intensity of the shifted bands (COMPETITION). Arrowheads indicate the position of the shifted bands. **D**) Two CERKL proteins moieties, Nter (amino acids 1–256) and Cter (amino acids 252–532) and the two negative controls from **C** were analyzed by EMSA using biotinylated mRNAs from COS-7 cells. Arrowhead indicates the position of the shifted band. **E**) Flag-tagged CERKL-WT or GFP proteins transiently expressed in HEK 293T cells were purified by immunoprecipitation, eluted with Flag peptide and then incubated with m^7^-GTP bound to Sepharose 4B beads (M7-GTP). Proteins bound to these beads were analyzed by Western blot with anti-Flag. As control, unconjugated Sepharose 4B beads (Seph) were used. Only CERKL-WT-Flag, but not GFP-Flag, binds to m^7^-GTP-Sepharose.

Until now, we have shown that CERKL is present in SGs, polysomes, PB and other mRNP compact particles. All these structures share mRNAs and proteins involved in mRNA metabolism, most of which also shift from the nucleus to the cytoplasm [18], [19]. To study if CERKL interacted with some of them, we performed immunoprecipitation experiments followed by liquid chromatography-tandem mass spectrometry. Table S1 shows a list of the twenty-seven identified proteins that interacted with CERKL. The majority (eighteen) corresponded to proteins involved in translation and protein folding, including PABP, HSP70 (known to assist in the folding of the nascent polypeptides), RPS3 and the translation initiation factor eIF3B. All these proteins, except eIF3B, also interacted with the Flag-tagged CERKL-C125W mutant (Fig. 4A). These interactions were subsequently corroborated by co-immunoprecipitation experiments followed by Western blots (Fig. 4B). Again, the protein eIF3B was shown to co-immunoprecipitate with CERKL-WT but not with the CERKL-C125W mutant.

To analyze the possibility that CERKL also binds to mRNA, RNA Electrophoretic Mobility Shift Assays (EMSA) were carried out using purified His-Maltose binding protein (MBP)-CERKL fusion proteins and biotinylated mRNAs isolated from human retina (Fig. 4C) or from COS-7 cells (Fig. S5). Some specific shifted bands were observed, indicating the formation of complexes between CERKL and some mRNAs. These complexes appeared at protein concentrations as low as 0.4 and 0.8 µM (with mRNAs from human retina and COS-7 cells, respectively) and contained CERKL as assessed by Western blot (data not shown). When bovine serum albumin (BSA) or MBP replaced CERKL, no shifted bands were produced. In addition, an excess of the non-biotinylated probe reduced the intensity of the shifted bands. Finally, the CERKL-mRNA binding could not be eliminated in a competition assay with tRNAs from *Saccharomyces cerevisiae* (data not shown). All these controls support the specificity of the CERKL-mRNA interaction.

To determine more accurately the CERKL domains responsible for the interaction with mRNA, two shorter peptides covering the full size protein were analyzed by EMSA, one comprising amino acids 1 to 256 and the other containing amino acids 252 to 532. Only the first peptide interacted with the mRNAs (Fig. 4D), indicating that the initial part of the protein is involved in this interaction. In addition, when possible sites of interaction with CERKL in the mRNAs were analyzed, we found that CERKL interacted with the 5′cap structure of mRNAs, since CERKL-Flag but not GFP-Flag was bound to 7-methylguanosine in an affinity assay with m7-GTP-Sepharose 4B beads (Fig. 4E). Moreover, since the C125W mutant also interacted with the 5′cap structure (data not shown), the cause of exclusion of C125W from SGs should not be due to a restricted binding but rather to a nucleus/cytoplasm shuttling defect.

### In the compact mRNPs most of the interactions of CERKL with the components of the translation machinery occur in an mRNA-dependent manner

**Figure 5:**
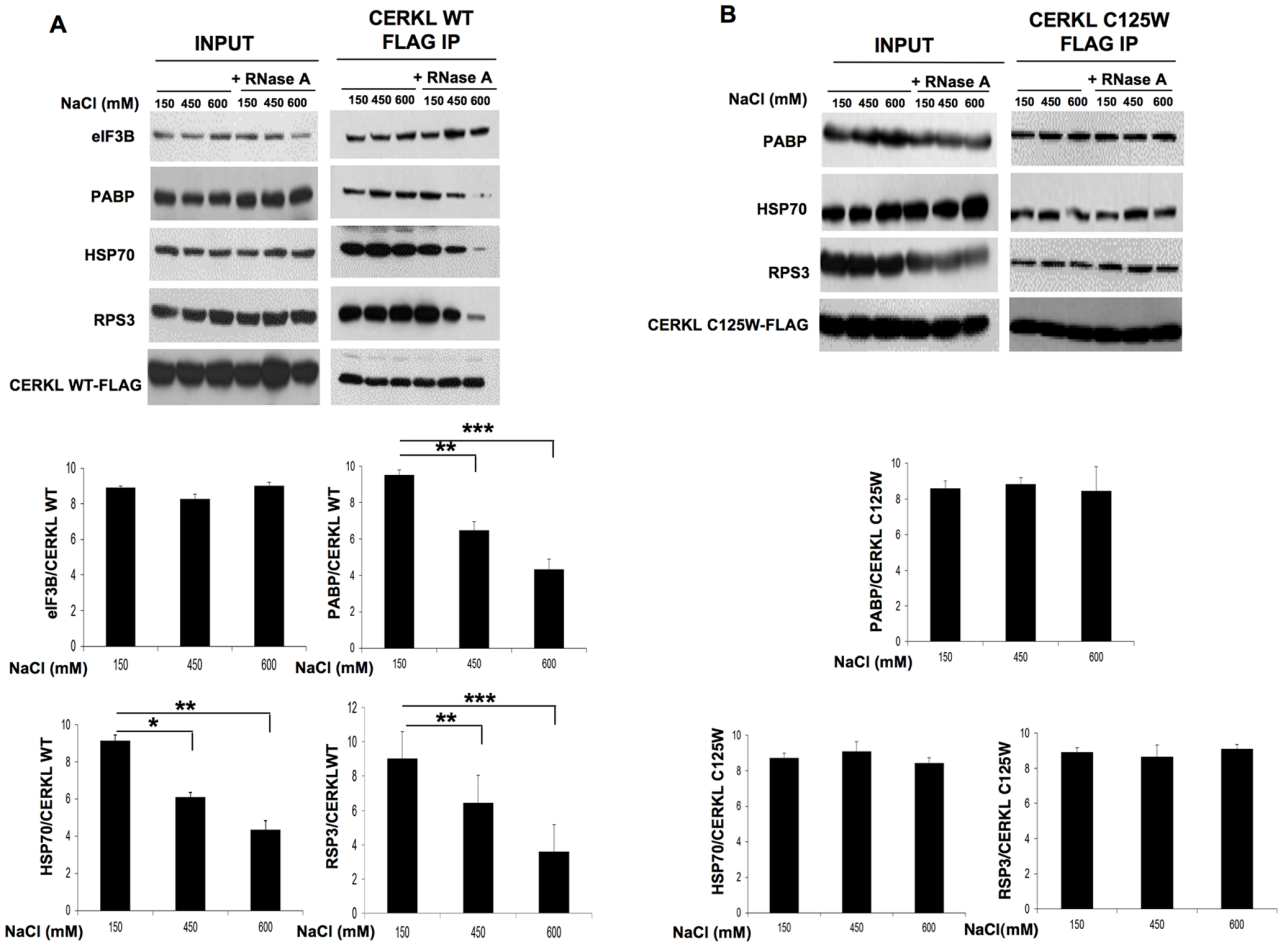
In the compact mRNPs, CERKL interacts with PABP, HSP70 and RPS3 in an mRNA-dependent manner. **A** and **B**) HEK-293T cells were transfected with CERKL-WT (**A**) or with CERKL-C125W mutant (**B**). After 48 h, cells were treated with 100 µg/ml cycloheximide and lysates were treated or not with RNase A (100 µg/mL) in the presence of three different concentrations of NaCl (150, 450 and 600 mM, as indicated). Then, the lysates were immunoprecipitated with anti-Flag M2 affinity beads. The co-immunoprecipitated proteins were analyzed by immunoblot using antibodies that recognize eIF3B (only in **A**), PABP, HSP70, RPS3 and Flag (to detect CERKL-WT and its C125W mutant). In the histograms below, the bands corresponding to the various proteins recovered after the Flag IP, in the presence of RNase A, were quantified with respect to CERKL-WT (**A**) or CERKL-C125W (**B**) in each lane. Values are the mean from 4 different experiments. Stars indicate statistically significant differences from the values in the presence of 150 mM NaCl (*p<0.05, **p<0.01 and ***p<0.005).

Concerning the four proteins of the translation machinery that were identified to be associated with CERKL (see Fig. 4A and B and Table S1), this association can occur by direct protein-protein interaction but also in an mRNA-dependent manner. Treatment of the lysates with RNase A did not affect these interactions (Fig. 5A). However, the results from Figure 3E and F showed that CERKL was associated with quite compact mRNPs, which only became sensitive to RNase A when they acquired a more relaxed conformation in the presence of high salt concentrations. Therefore, we carried out the immunoprecipitation experiments of Flag-tagged CERKL after treating the cell lysates with RNase A at different salt concentrations (150, 450 and 600 mM NaCl) before immunoprecipitation. As shown in Figure 5A, in the presence of high salt concentrations RNase A affected the interaction of CERKL with PABP, HSP70 and RPS3. These effects were not observed with the CERKL-C125W mutant (Fig. 5B). In contrast to PABP, HSP70 and RPS3, the interaction of CERKL-WT with eIF3B was not lost under high salt conditions (Fig. 5A, upper gel). Therefore, except for eIF3B, in the compact mRNP particles the interaction of CERKL with the three remaining proteins here investigated occurred in an mRNA-dependent manner.

### Compact CERKL-mRNPs interact with microtubules

**Figure 6:**
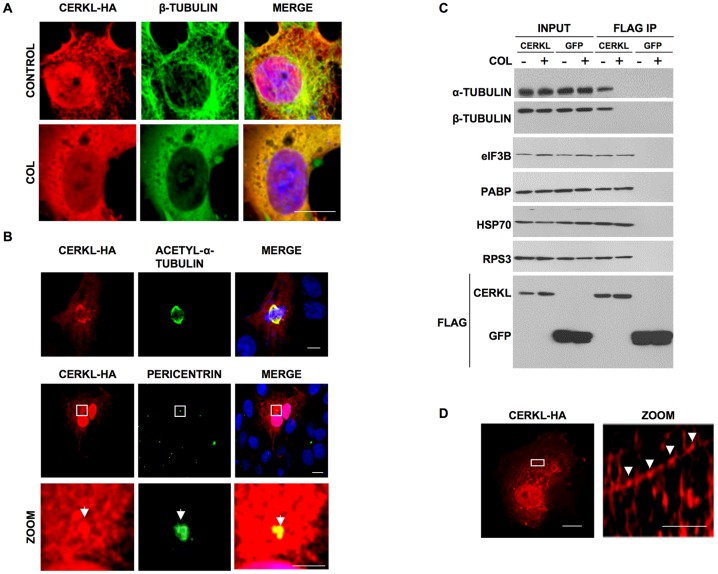
CERKL interacts with microtubules. **A**) COS-7 cells overexpressing CERKL were fixed with 2% paraformaldehyde and the localizations of CERKL (HA) and ß-tubulin were compared by immunofluorescence. As control, cells were treated with 1 µg/ml colchicine (COL) for 2 h to disrupt microtubules. Bar: 10 µm. **B**) CERKL colocalizes with microtubule-related structures. COS-7 transfected cells were fixed with 2% paraformaldehyde and the localization of CERKL (HA) was compared by immunofluorescence with that of acetyl-α-tubulin (upper panels) and the centrosomal protein pericentrin (lower panels, arrowhead) using specific antibodies. Images at higher magnification of the rectangles in the upper row are shown below. All bars: 10 µm. **C**) Immunoblot of alpha and ß-tubulin, eIF3B, PABP, HSP70 and RPS3 proteins co-immunoprecipitating with CERKL-Flag or GFP-Flag in the presence (+) or not (−) of 1 µg/ml colchicine (COL), as indicated. Bottom, CERKL and GFP in the various lanes using anti-Flag. **D**) COS-7 cells transfected with CERKL-HA were fixed with 2% paraformaldehyde and CERKL (HA) was localized by immunofluorescence. Images at higher magnification of the rectangles are shown on the right. Arrowheads indicate the particles containing CERKL. Bars: 10 µm (original image) and 2 µm (zoom).

Alpha- and beta-tubulin were among the proteins identified in the immunoprecipitation experiments with CERKL (Fig. 4A and table S1). This association was further corroborated by immunofluorescence (Fig. 6A). In addition, when the cells were fixed with 2% instead of 4% paraformaldehyde, a filamentous appearance of CERKL was noticed that was disrupted by a colchicine treatment, indicating that this distribution was due to its interaction with microtubules (Fig. 6A, lower panels). CERKL also co-localized with other structures related to microtubules, such as the mitotic spindle or the centrosome (Fig. 6B). The interaction of CERKL with α- and β-tubulin was also confirmed by co-immunoprecipitation (Fig. 6C). To determine whether the interaction of CERKL with microtubules was necessary for the formation of the CERKL complex with the proteins of the translation machinery, we performed co-immunoprecipitation experiments in the presence or absence of colchicine. As expected, the interaction of CERKL with α- and β-tubulin was lost after colchicine treatment, confirming that it was bound to microtubules (Fig. 6C). However, the interaction of CERKL with the different proteins of the translation machinery was not affected by the colchicine treatment (Fig. 6C). According to these data, we can conclude that the interaction of CERKL with the proteins of the translation machinery does not require the integrity of the microtubules and, therefore, that the complex binds to them after it has been formed.

**Figure 7:**
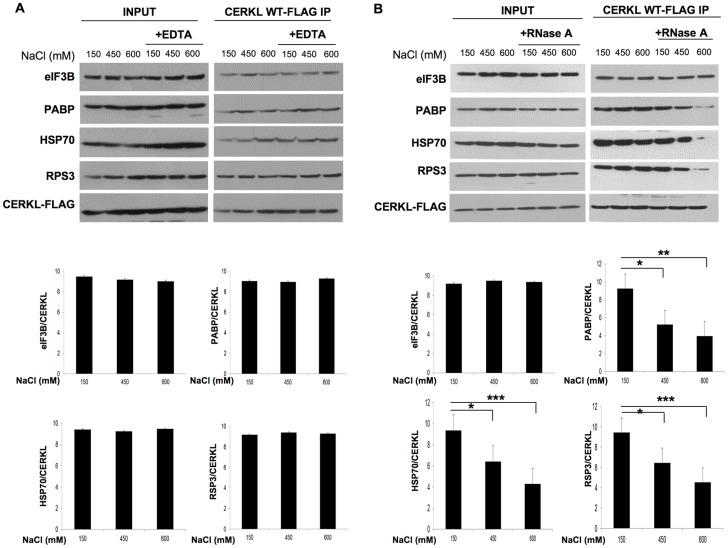
The compact CERKL-mRNP complexes interact with microtubules. HEK-293T cells were transfected with CERKL-WT and after 48 h a cytoskeletal fraction was isolated from the cells as described in Materials and Methods. Fractions were treated with 15 mM EDTA (**A**) or 100 µg/ml RNase A (**B**) in the presence of 150, 450 and 600 mM NaCl, as shown in the figure, and then subjected to immunoprecipitation with anti-Flag M2 affinity beads. The co-immunoprecipitated proteins were analyzed by immunoblot using antibodies that recognize eIF3B, PABP, HSP70, RPS3 and Flag (for CERKL). In the histograms below, the bands corresponding to the various proteins recovered after the Flag IP, in the presence of RNase A, were quantified with respect to CERKL-WT (**A**) or to CERKL-C125W (**B**) in each lane. Values are the mean from 4 different experiments. Stars indicate statistically significant differences from the values in the presence of 150 mM NaCl (*p<0.05, **p<0.01 and ***p<0.005).

When cells are fixed with 2% paraformaldehyde, CERKL can be also observed in the form of particles of 100–250 nm diameter (Fig. 6D). To verify that these complexes actually bind to microtubules, we immunoprecipitated CERKL from a purified cytoskeletal fraction (Fig. S6) and confirmed the interaction of CERKL with eIF3B, PABP, HSP70 and RPS3 (Fig. 7). In this fraction, the interaction of CERKL with these proteins was not sensitive to EDTA (Fig. 7A) and, except for eIF3B, it was only sensitive to RNase A at a high salt concentration (Fig. 7B). These results indicate that CERKL and the other proteins form compact mRNP complexes independently of 80S ribosomes and, therefore, that the mRNAs in these complexes are untranslated.

### CERKL colocalizes with microtubules and components of the translation machinery in neurites of differentiated human neuroblastoma cells

**Figure 8:**
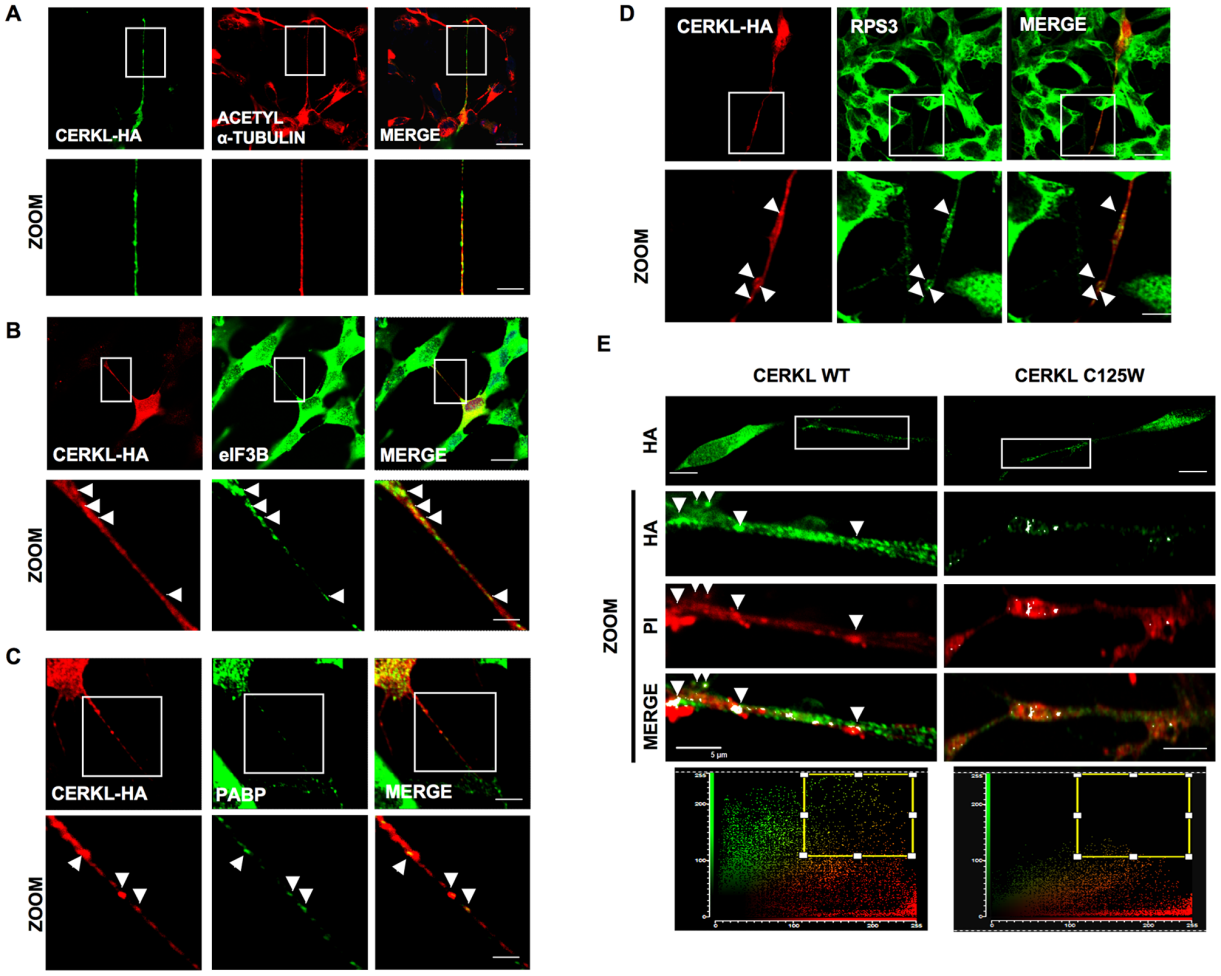
CERKL localization in differentiated SH-SY5Y neural cells. **A**) SH-SY5Y cells were transfected with CERKL-HA and after 48 h cells were differentiated with 10 µM retinoic acid. The distribution of CERKL and acetyl-α-tubulin were compared after 36 h by immunofluorescence. Images at higher magnification of the rectangles in the upper row are shown below. **B**–**D**) SH-SY5Y were transfected and differentiated as in **A**. Images show colocalization by immunofluorescence of CERKL (HA) with eIF3B (**B**), PABP (**C**) and RPS3 (**D**) in differentiated SH-SY5Y cells. Images at higher magnification of the rectangles in the upper row are shown below. **E**) Colocalization of CERKL-WT and CERKL-C125W (HA) with RNA (propidium iodide staining, PI) in differentiated SH-SY5Y cells. Arrowheads show colocalization in particulated structures. The scatter diagrams below show colocalization dots. Bars: 10 µm (original images) and 5 µm (zoom).

We have observed that CERKL associates with translationally inactive compact mRNPs that bind to microtubules. We next analyzed if this also happens in human differentiated SH-SY5Y neuroblastoma cells because these cells present more structural similarities with retinal ganglionar cells and photoreceptors, which are the main targets of the CERKL defects and are also the cells where this protein is more abundant. In the neurites of these cells, CERKL colocalized with acetyl-alpha-tubulin, thus indicating again its association with microtubules (Fig. 8A). In addition, CERKL also presented a particulated distribution that colocalized with eIF3B, PABP and RPS3 (Fig. 8B–D). The fact that these particles also contained RNA (propidium iodide staining in Fig. 8E, left panels) indicated that the CERKL-containing mRNPs are also found in neurites in differentiated cells. Noticeably, the CERKL-C125W mutant (Fig. 8E, right panels) neither presented this particulated distribution nor colocalized with RNA, confirming its inability to form mRNPs.

## Discussion

Within the last two and a half decades, technological advances have led to a progress in gene identification of retinal diseases. However, the function of a high number of the over hundred retinal diseases and fifty RP genes known remains to be characterized. Functional studies have been hampered mainly by the lack of suitable animal and cellular models, the intracellular complexity and specific metabolic demands of the retinal cells and the extensive genetic, allelic and clinical heterogeneity associated with the majority of retinal degenerative disorders. Within this context, *CERKL*, an autosomal recessive RP and CRD -causing gene, stands among the genes whose function awaits characterization. In fact, repeated attempts by several groups to assign a ceramide kinase function to CERKL have proved fruitless [8], [9], [10], [12].

Therefore and to gain some insight into CERKL function we investigated here in detail the subcellular localization and the interaction partners of the CERKL full length protein isoform (CERKLa). The protein was localized both in the nucleus and in the cytoplasm of various cell types, in agreement with other studies [8], [9], [10], [20]. Although the CERKL distribution appeared to be mainly homogeneous, some aggregates became apparent in the cytoplasm of transiently transfected cells. A systematic analysis in cultured cells using different markers of various cell compartments revealed that CERKL aggregates colocalized with SGs, which are cytoplasmic complexes of mRNAs and mRNA-binding proteins such as those that regulate mRNA translation and stability [21]. These granules are induced by stress conditions and provide the cells with a mechanism to stop protein synthesis and promote a quick recovery of proteostasis when the stress disappears. Targeting to SGs under stress conditions would agree with CERKL protective role from apoptosis induced by oxidative stress [10]. In addition to SGs, CERKL was also found associated with P-bodies, which are involved in mRNA degradation and are in dynamic equilibrium with SGs [22]. Also in support to a CERKL contribution to stress response and protection of photoreceptors is the punctuated CERKL labeling noticed after a light stress in the outer nuclear layer of rat photoreceptors [4].

The CERKL-C125W mutant, which does not enter the nucleus, is not found in SGs. In fact, the localization of wild type CERKL to SGs seems to depend on nuclear import/export of the protein and on mRNA transcription. Therefore, we reasoned that CERKL has a role in the nucleus before it associates with SGs, probably related to RNA transport. In fact, CERKL subcellular localization studies have shown strong accumulation in the nucleoli [10]. Whether this is related to particular protein isoforms or depends on the cellular state deserves further study. Actually, there are other genes mutated in RP that are involved in RNA metabolism at the nucleus, such as the spliceosome components PRPF31, PRPF8 and PRPF3, or the RNA splicing factor RP9 [1], [23]. Recently, in age-related macular degeneration, SFRS10 has been reported to regulate alternative splicing of stress response genes under hypoxic conditions [24].

In the cytosol, apart from the localization of CERKL in aggregates, the protein was mostly found diffusely distributed. This was particularly evident in HeLa cells stably expressing this protein. Since SGs are in dynamic equilibrium with polysomes, we analyzed whether this diffuse localization of CERKL could correspond to its binding to polysomes. Using sucrose gradients, CERKL localized to the polysomal pellet and to a greater extent in the soluble fractions that contained postpolysomal mRNPs. Contrary to polysomes, the association of CERKL to these fractions was not sensitive to EDTA or puromycin and was only sensitive to RNase A at high salt concentrations. Therefore, it appears that CERKL in these fractions is associated with quite compact mRNPs.

The association of CERKL to mRNPs can occur by protein-RNA and protein-protein interactions. Our data show that CERKL directly interacts with mRNA through its N-terminal region and that this link involves the 5′cap structure of mRNA. Since in the proteomic analysis (table S1) we did not observe any interaction between CERKL and the two mammalian proteins known to bind directly to the 5′cap structure, elF4E and nCBC [25], and since eIF4E was not detected in immunoprecipitation assays (data not shown), the CERKL-mRNA association is probably direct. However, we cannot exclude an interaction with a yet unidentified component of the 5′cap structure. In addition, CERKL was found to bind to proteins of the translation machinery, such as PABP, eIF3B, HSP70 and the ribosomal protein RPS3, all of which are found in SGs and in other mRNPs, such as neuronal and transport granules [21], [26], [27], [28], [29]. Except for eIF3B, the interaction of CERKL with these proteins was mRNA-dependent, mostly forming compact RNPs. Interestingly, the CERKL-C125W mutant did not interact with eIF3B and its binding to the other proteins was not sensitive to RNase A even at high salt concentrations, indicating the absence of RNA in these complexes. This together with the restricted nuclear/cytoplasmic shutling ability shown for this mutant could explain why it is not found in Sgs.

CERKL-containing compact mRNPs were found associated with microtubules and in the cytoskeletal fraction. In fact, CERKL contains a PH domain that is also present in proteins that bind to microtubules [30]. This association could be found here in distal compartments of differentiated neural cells. These results suggest a role of this protein in the transport of the mRNAs present in these structures, although this needs to be further investigated. In this regard, it is known that a compact packaging of mRNAs into mRNPs is essential to protect the mRNAs and to guarantee their survival until translation occurs [31]. The assembly of these mRNPs starts while the mRNA is still being transcribed. This explains the requirements of an active transcription, the nuclear import/export cycle of CERKL and why the CERKL C125W mutant is unable to form these complexes. Although we have highlighted a new cellular role of CERKL and shown that a pathological mutant lacks these features, further studies are needed to substantiate the *in vivo* role of CERKL variants in the retina, focusing on the relationship between the protein isoforms, their intracellular localization and the disease phenotype.

In summary, our data unveil a novel cellular localization associated with hereditary retinal disorders. CERKLa is a new component of a group of compact mRNPs containing proteins of the translation machinery and untranslated mRNAs. If these mRNPs include mRNAs relevant for the normal function of retinal cells, currently being studied, this would provide the basis to understand the physiological disturbances caused by the different mutations in this protein and the mechanism of retinal degeneration in health and disease, and unveil new pathways to identify molecular targets for therapy.

## Materials and Methods

### Expression plasmids and transfections

For transfection assays, the *CERKL* cDNA transcript variant 1 (NM_201548.4) was cloned into pEGFP-C2 (Addgene) (CERKL-GFP) and into modified versions of pcDNA (Clontech Laboratories, Inc.), which add a C-terminal hemagglutinin (CERKL-HA) or a Flag (CERKL-Flag) tag. The mutant encoding amino acids 1 to 256 tagged with HA was cloned into pcDNA (CERKL 1–256). All constructs were generated using standard recombinant DNA techniques. A plasmid expressing Flag-GFP was kindly provided by Dr. A.T. Ting (Mount Sinai School of Medicine, NY, USA). A CERKL-C125W point mutant was generated using the QuikChange XL site-directed mutagenesis kit (Stratagene/Agilent Technologies), according to the manufacturer's instructions. To produce recombinant MBP-CERKL fusion proteins in *E. coli*, the cDNA encoding the full length CERKL protein or its amino acids 1–256 and 252–532 were inserted into the pDEST-His-MBP vector (Addgene) using the Gateway recombination cloning kit (Invitrogen Life Technologies). All constructs were confirmed by sequence analysis.

### Cell culture and stress treatments

COS-7, HEK-293T, HeLa (ECACC, Salisbury, UK), 661W [32] and SH-SY5Y (ATCC, LGC Standards, Barcelona, Spain) cells were grown in DMEM with 4 mM L-glutamine supplemented with 10% fetal bovine serum, 100 U/mL penicillin and 100 µg/mL streptomycin (Invitrogen Life Technologies). As for 661W cells, the medium was supplemented with 0.002% 2-mercaptoethanol. HeLa cells stably expressing CERKL were generated by transfection with pcDNA-CERKL-HA and positive clones were selected with G418 (Invivo Gen). The human neuroblastoma cell line SH-SY5Y was transfected with CERKL-HA and after 48 h the cells were differentiated by incubating them in DMEM supplemented with 0.5% fetal bovine serum and 10 µM retinoic acid (Sigma-Aldrich). Experiments were performed after thirty-six additional hours. For transfection, we used FuGene HD (Roche Diagnostics) and Metafectene Pro (Biontex) following the manufacturer's instructions. All cells were maintained in a sterile humidified environment at 37°C in a 5% CO_2_ atmosphere. Where indicated, cells were treated, after 36 or 48 h of transfection, with 40 nM leptomycin B, 1 µg/mL actinomycin D, 100 µg/mL cycloheximide or 100 µg/mL alpha-amanitin (all from Sigma-Aldrich) for the indicated times. For induction of stress, cells were incubated for 30 min either at 44°C (heat shock) or with 0.5 mM sodium arsenite (Sigma-Aldrich). In some of the samples, cell stress was induced 15 min before the cycloheximide treatment.

### Immunofluorescence, fluorescence *in situ* hybridization and confocal microscopy

Immunolocalization was performed as previously described [10], [33] incubating the cells with anti-HA mouse monoclonal antibody (1∶100, Abcam) and with either of the following antibodies (all from rabbit): anti-calnexin, anti-GM130 and anti-TGN48 (1∶100, BD Biosciences), anti-EEA1, anti-EDC4, anti-Rab11a, anti-clathrin, anti-LAMP1, anti-eIF4E, anti-PABP, anti-G3BP 1, anti-AFDP, anti-EDC4, anti-pericentrin and anti-β-tubulin (1∶100, Abcam), anti-LBPA (1∶100, Echelon Biosciences), anti-LC3 (1∶200, Nanotools), anti-p62 and anti-ubiquitinated proteins (1∶200, Enzo Life Sciencies). Upon washing, cells were incubated, as appropriate, with AlexaFluor 488-conjugated anti-rabbit or AlexaFluor 546-conjugated anti-mouse secondary antibodies (1∶300, Molecular Probes). For α- and β-tubulin staining, cells were fixed with 2% paraformaldehyde in PBS. To disrupt microtubules, cells were incubated for 2 h with 1 µg/ml colchicine (Sigma-Aldrich). Controls were carried out in the absence of the primary antibodies. In colocalization experiments, cross-binding controls using the two secondary antibodies with each primary antibody were also included. Mitochondria were labeled by adding reduced MitoTracker Orange (Invitrogen Life Technologies) to the cell culture medium at a final concentration of 500 nM for 45 min. Lipid droplets were stained with BODIPY 493 (Invitrogen Life Technologies). Slides were counterstained with 1∶5,000 DAPI nuclear blue dye (Roche Diagnostics) in PBS for 15 min. Fluorescence *in situ* hybridization (FISH) with biotinylated oligo(dT) (Sigma-Aldrich) was carried out as previously described [34].

All preparations were mounted in Fluoprep medium (BioMérieux) or in FluorSave reagent (Calbiochem-Merck4Biosciences) and images were obtained with a confocal microscope (SP2; Leica Microsystems) or with an Apotome-equipped Axio Observer Z1 microscope (Carl Zeiss AG).

### Isolation of polysomes

Polysomes were isolated as described elsewhere with some modifications [35], [36]. After 30 min treatment with cycloheximide, HeLa cells stably transfected with CERKL-HA were collected and homogenised in 0.25 M sucrose, 25 mM NaCl, 5 mM MgCl_2_, 50 mM Tris pH 7.4, for 7 min at 200 psi in a N_2_ cavitation pump (Parr Instruments Co.), followed by five passages through a 25G-gauge needle. The homogenates were centrifuged at 10,000 g for 10 min. In separate control experiments, 15 mM EDTA or 100 µg/ml RNase A (Sigma-Aldrich) were added to disrupt polysomes. The supernatants were applied onto the top of a discontinuous sucrose gradient (0.5, 1.3, 1.7 and 2.1 M sucrose plus 5 mM MgCl_2_ and 1 mM dithiothreitol) and centrifuged at 53,000 rpm for 2 h in a 70.1 Ti rotor (Beckman). Seven fractions (about 1 ml) were collected, precipitated with 10% trichloroacetic acid and used, together with the ultracentrifugation pellet, for protein analysis by Western blot. A second continuous sucrose gradient (0.3–1.5 M) was employed to further analyze separately the pellet and a mix of two of the upper fractions of the discontinuous gradient by centrifugation at 28,000 rpm for 4 h in an SW40 rotor (Beckman). Ten or eleven fractions and the pellet were analyzed as above.

### Immunoprecipitation

Immunoprecipitation of Flag-tagged proteins was carried out essentially as previously described [37]. Where indicated, cells were treated with 100 µg/ml cycloheximide prior to lysis in 1% Triton X-100, 150 mM NaCl, 20 mM Tris, pH 7.4, containing 0.1 mM leupeptin, 1 mM phenyl methane sulfonyl fluoride, 10 mM pepstatin A, 50 mM NaF, 30 mM Na_4_P_2_O_7_ (lysis buffer). The resulting extracts were sonicated (output: 9 watts) 3 times (20 seconds each) with a Microson Ultrasonic cell disruptor, incubated next with albumin conjugated agarose beads and centrifuged at 12,000 g for 15 min. The pre-cleared lysates were then incubated with M2 Flag beads (Sigma-Aldrich) for 1 h at 4°C and, upon extensively washing, immunoprecipitated proteins were eluted with 300 µg/mL Flag peptide (Sigma-Aldrich) for 1 h at 4°C. Treatments with EDTA (15–30 mM), puromycin (1 mM, Sigma-Aldrich) or RNase A (100 µg/mL) at the indicated salt concentrations were carried out where indicated after pre-clearing the samples with the agarose beads.

### Liquid chromatography-tandem mass spectrometry

CERKL-Flag, CERKL-C125W-Flag and GFP-Flag were immunoprecipitated as described above and proteins were separated in 8% and 20% polyacrylamide gels. All following treatments were conducted at the CIPF proteomic core facility. After silver staining, differential bands were excised and digested with sequencing grade trypsin as described elsewhere [38]. The final peptide solution was concentrated and analyzed by mass spectrometry (Nano ESQqTOF, AB SCIEX, Framingham, MA). Protein identification was carried out using the ProteinPilot (AB SCIEX) search engine on the ExPasy protein database. The accuracy of proteins identification was considered when at least three peptides were identified with a minimum overall MASCOT score >50.

### RNA electrophoretic mobility shift assays

His-MBP-CERKL fusion proteins were produced in *E. coli* and purified with the His SpinTrap kit (GE Healthcare). Messenger RNA was purified from total RNA from COS-7 cells and from human retina using the Oligotex mRNA kit (Qiagen) and biotinylated using the RNA 3′ biotinylation kit (Thermo Fisher Scientific). EMSA was conducted using LightShift Chemiluminescent RNA EMSA kit (Thermo Fisher Scientific) according to the manufacturer's protocol. BSA was used as a negative control. The protein-probes complexes were resolved in a 6% polyacrylamide native gel and transferred to a Hybond-N^+^ Nylon membrane (GE Healthcare). Migration of biotin-labeled probes was detected on an ImageQuant™ LAS 4000 biomolecular imager (GE Healthcare) using streptavidin-horseradish peroxidase conjugates and chemiluminescent substrates for biotin recognition.

### 7-methylguanosine affinity assay

Immunoprecipitated CERKL-Flag or GFP-Flag were incubated overnight at 4°C with m^7^-GTP-Sepharose or Sepharose-4B beads (GE Healthcare Life Sciences) in the immunoprecipitation lysis buffer. Sepharose beads were centrifuged and washed three times in the same buffer containing 450 mM NaCl. Proteins were eluted by incubating the beads in SDS-PAGE loading buffer at 70°C for 20 min, separated from the beads using the Pierce Spin Cups with cellulose acetate filter (Thermo Fisher Scientific) and then analyzed by Western blot.

### Isolation of a cytoskeletal fraction

A cytoskeletal fraction was isolated with the Subcellular Protein Fractionation Kit (Thermo Scientific) according to the manufacturer's instructions. The cytoskeletal pellet was resuspended in the immunoprecipitation lysis buffer, sonicated 2 times (output 6 watts, 20 seconds each) with a Microson Ultrasonic cell disruptor and centrifuged at 12,000×g for 10 h at 4°C. Supernatants were collected and used for subsequent analysis.

### Other procedures

Western blot was carried out essentially as described [39], using the following primary antibodies: anti-HSP70, anti-eIF3B, anti-HA, anti-PABP, anti-ribosomal proteins S3, S6 and L26, anti-RNase A, anti α- and β-tubulin (all from Abcam), anti-Flag and anti-actin (Sigma-Aldrich), followed by the corresponding horseradish peroxidase-conjugated secondary antibodies (all from Sigma-Aldrich). Protein concentration was determined using the BCA Protein Assay Kit (Thermo Fisher Scientific) according to the manufacturer's instructions. Comparisons among different conditions, after calculating mean and SD values, were by Student's *t* test. Differences were considered significant at *P<0.05, **P<0.01 and ***P<0.005.

## Supporting Information

Figure S1 **CERKL does not colocalize with various organelle markers.** The localization of CERKL in COS-7 cells overexpressing CERKL-HA was compared to that of: **A**) calnexin (endoplasmic reticulum); **B** and **C**) GM130 and TGN48 (*cis* and *trans* Golgi, respectively); **D**) Mitotracker (mitochondria); **E, F** and **G**) EEA1, LBPA and Rab11a (early, late and recycling endosomes, respectively); **H**) clathrin (clathrin-coated vesicles); **I**) LAMP1 (lysosomes), and **J**) LC3 (autophagosomes). No colocalization with any of these organelle markers was found and only a partial distribution of CERKL around the endoplasmic reticulum was observed (in **A**). Bar: 10 µm.
(TIF)

Figure S2 **Localization of CERKL to stress granules is lost in the presence of cycloheximide.** COS-7 cells transfected with CERKL-HA were incubated with 500 µM sodium arsenite (SA) for 1 h. In the last 30 min of incubation, 100 µg/mL cycloheximide (CHX) was added. The localizations of CERKL (HA) and PABP were compared by immunofluorescence. Bar: 10 µm.
(TIF)

Figure S3 **GFP does not colocalize with stress granules.** COS-7 cells were transfected with GFP and its localization was compared to that of PABP. Immunofluorescence images show no colocalization of GFP with the marker of stress granules. Bar: 10 µm.
(TIF)

Figure S4 **CERKL localizes to stress granules in HeLa cells under stress conditions.** **A**) Colocalization experiments of overexpressed CERKL-HA in HeLa cells, untreated (MOCK) or treated with 500 µM sodium arsenite (SA) for 30 min. In the presence of SA, CERKL colocalizes with eIF4E, a marker of stress granules. Images at higher magnification of the rectangles are shown on the right (ZOOM). All bars: 10 µm. **B**) CERKL-HA also colocalizes with another marker of stress granules (PABP) in HeLa cells, when treated with SA as above or when subjected to a 44°C heat shock for 30 min (two middle panels). This colocalization is lost after cycloheximide (CHX) treatment (lower panel). Images at higher magnification of the rectangles are shown on the right. All bars: 10 µm. **C**) CHX does not affect the total amount of CERKL in the cells. HeLa cells overexpressing CERKL-HA were incubated at 37°C, 44°C or 44°C plus 100 µg/ml cycloheximide (+ CHX) and, after 30 min, CERKL levels in cell lysates were analyzed by Western blot with antibodies that recognize HA (upper panel) or, as a loading control, tubulin (lower panel). The numbers below indicate the relative amount of CERKL with respect to tubulin in each lane.
(TIF)

Figure S5 **CERKL interacts with mRNAs.** CERKL protein was purified and serial dilutions of the protein were incubated with biotinylated mRNAs from COS-7 cells. Specific shifted bands were observed (arrowheads) at protein concentrations as low as 0.8 µM in COS-7 mRNAs. Bovine serum albumin (first lane, CONTROL) and His-maltose binding protein (second lane, 0) were used as negative controls and addition of an excess of non-biotinylated probes reduced the intensity of the shifted bands (last lane, COMPETITION).
(TIF)

Figure S6 **Isolation of a cytoskeletal fraction.** Fractionation of HEK-293T cells to obtain a cytoskeletal fraction (SK) was carried out as described in Materials and Methods. Purity of each fraction was analyzed by Western blot using antibodies that recognize HSP70 and glyceraldehyde-3-phosphate dehydrogenase (GAPDH) as markers of the soluble fraction (S), calnexin and porin as markers of the membrane fraction (M), lamin and histone H3 as markers of nuclei (N) and chromatin (C), respectively, and acetyl-α-tubulin as a microtubule marker. Vimentin (intermediate filaments marker) was used as a control. H: total homogenate.
(TIF)

Table S1 **CERKL interacting proteins.**
(DOC)
