# Supplementary figures and images for: CERKL, a Retinal Disease Gene, Encodes an mRNA-Binding Protein That Localizes in Compact and Untranslated mRNPs Associated with Microtubules
Source: PLoS One. 2014 Feb 3;9(2):e87898. doi: 10.1371/journal.pone.0087898 (PMC3912138; doi:10.1371/journal.pone.0087898)

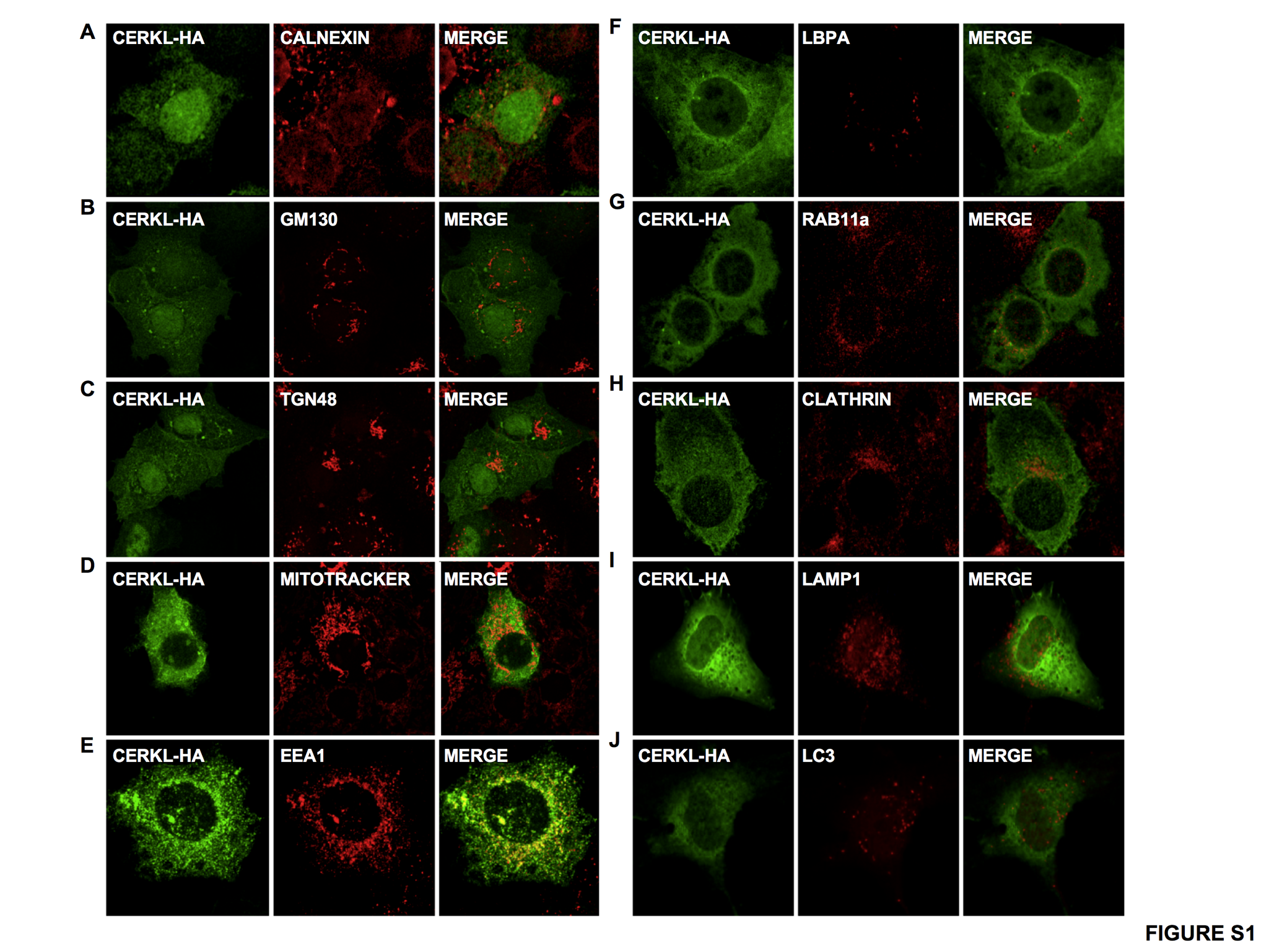

Supplement: Figure S1 — CERKL does not colocalize with various organelle markers. The localization of CERKL in COS-7 cells overexpressing CERKL-HA was compared to that of: A) calnexin (endoplasmic reticulum); B and C) GM130 and TGN48 (cis and trans Golgi, respectively); D) Mitotracker (mitochondria); E, F and G) EEA1, LBPA and Rab11a (early, late and recycling endosomes, respectively); H) clathrin (clathrin-coated vesicles); I) LAMP1 (lysosomes), and J) LC3 (autophagosomes). No colocalization with any of these organelle markers was found and only a partial distribution of CERKL around the endoplasmic reticulum was observed (in A). Bar: 10 µm. (TIF) [file pone.0087898.s001.tif]

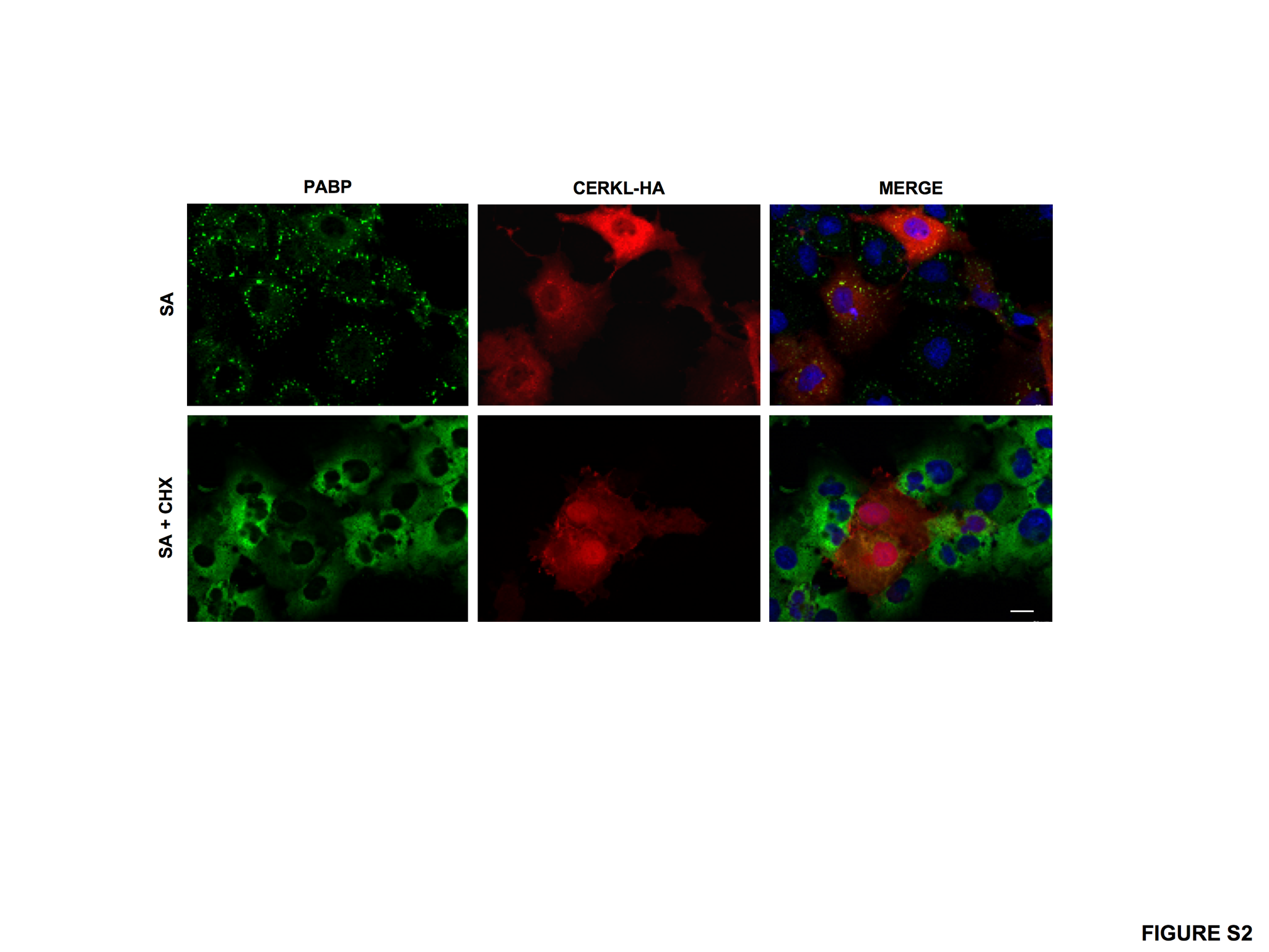

Supplement: Figure S2 — Localization of CERKL to stress granules is lost in the presence of cycloheximide. COS-7 cells transfected with CERKL-HA were incubated with 500 µM sodium arsenite (SA) for 1 h. In the last 30 min of incubation, 100 µg/mL cycloheximide (CHX) was added. The localizations of CERKL (HA) and PABP were compared by immunofluorescence. Bar: 10 µm. (TIF) [file pone.0087898.s002.tif]

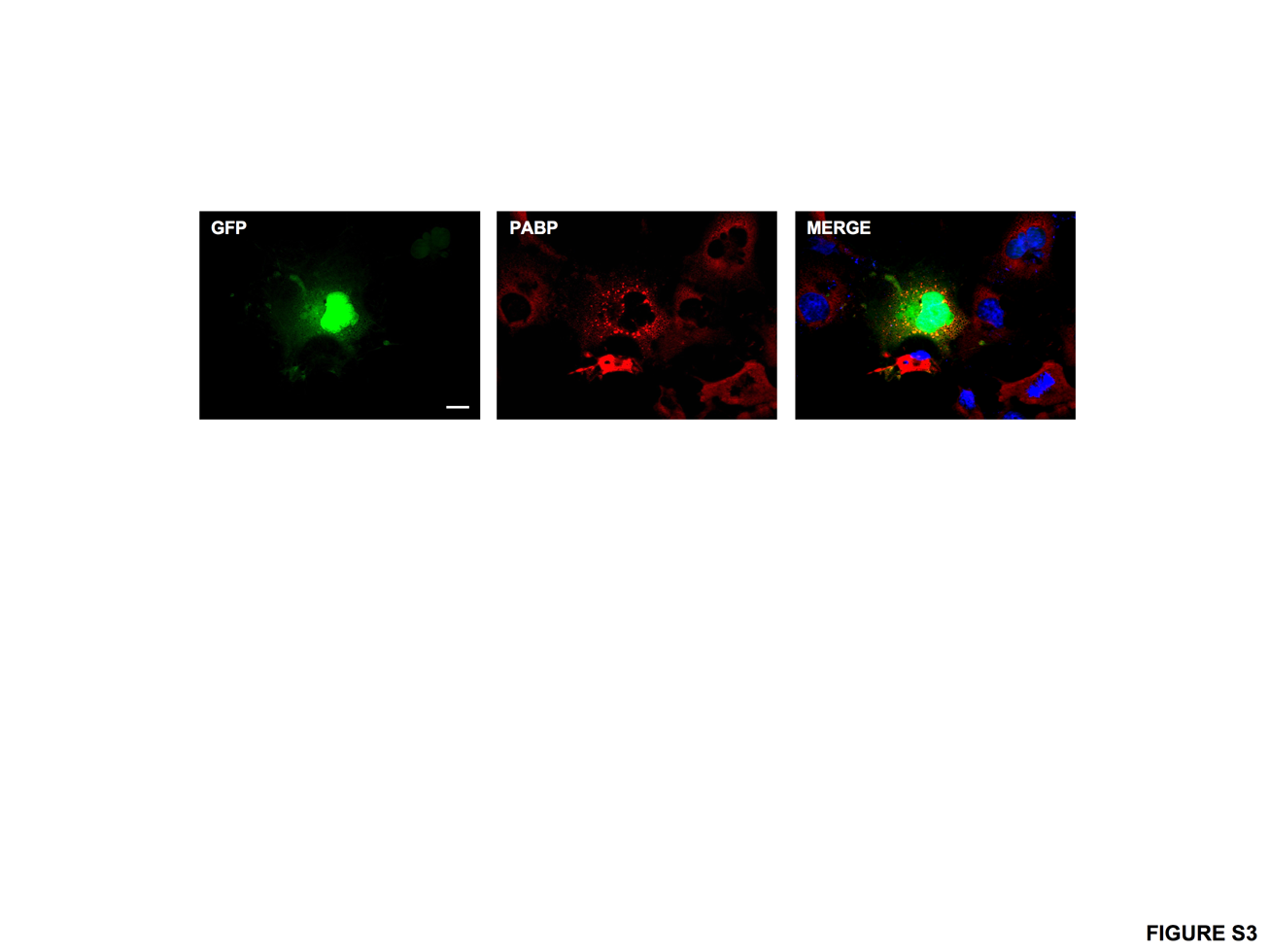

Supplement: Figure S3 — GFP does not colocalize with stress granules. COS-7 cells were transfected with GFP and its localization was compared to that of PABP. Immunofluorescence images show no colocalization of GFP with the marker of stress granules. Bar: 10 µm. (TIF) [file pone.0087898.s003.tif]

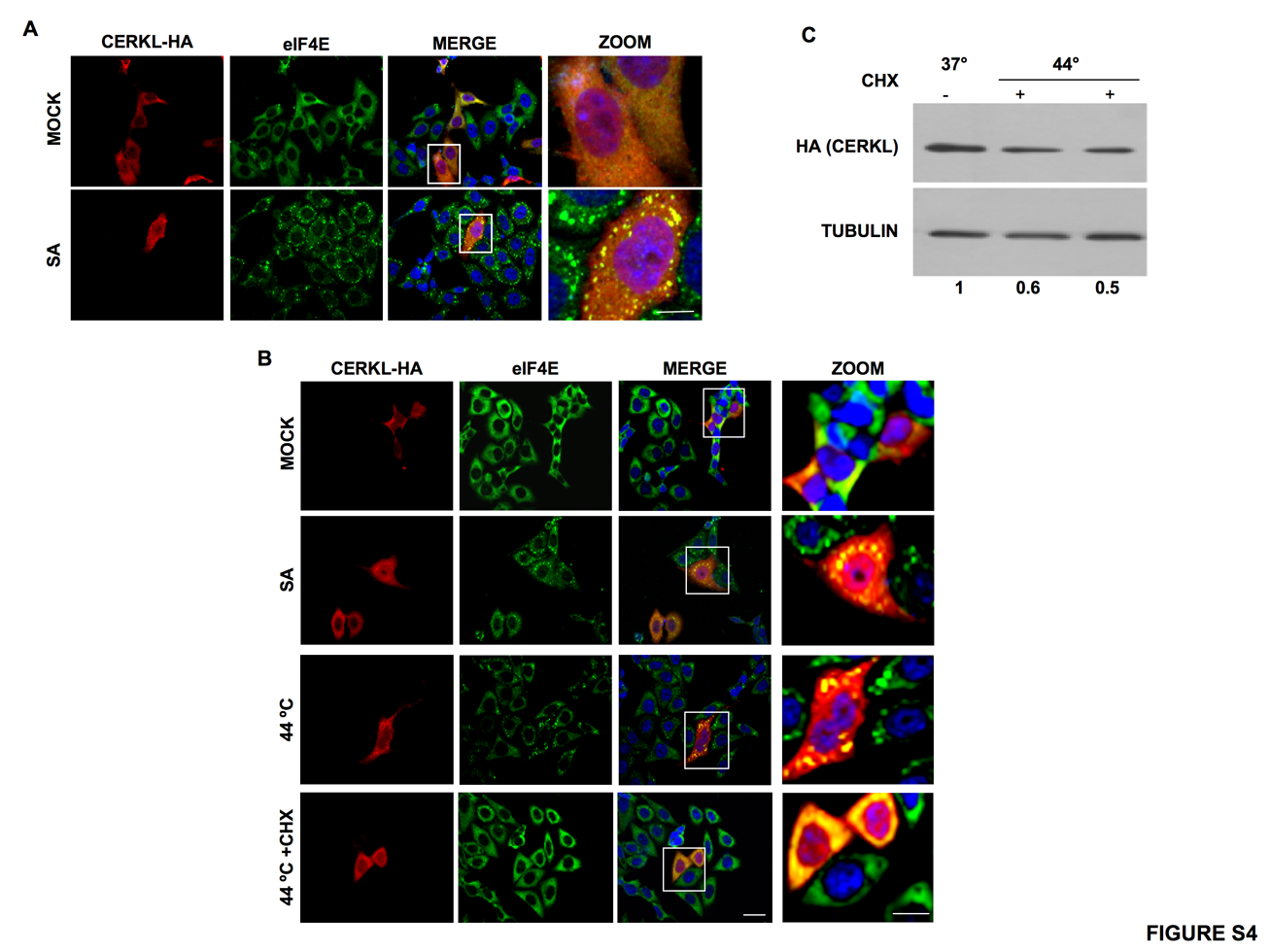

Supplement: Figure S4 — CERKL localizes to stress granules in HeLa cells under stress conditions. A) Colocalization experiments of overexpressed CERKL-HA in HeLa cells, untreated (MOCK) or treated with 500 µM sodium arsenite (SA) for 30 min. In the presence of SA, CERKL colocalizes with eIF4E, a marker of stress granules. Images at higher magnification of the rectangles are shown on the right (ZOOM). All bars: 10 µm. B) CERKL-HA also colocalizes with another marker of stress granules (PABP) in HeLa cells, when treated with SA as above or when subjected to a 44°C heat shock for 30 min (two middle panels). This colocalization is lost after cycloheximide (CHX) treatment (lower panel). Images at higher magnification of the rectangles are shown on the right. All bars: 10 µm. C) CHX does not affect the total amount of CERKL in the cells. HeLa cells overexpressing CERKL-HA were incubated at 37°C, 44°C or 44°C plus 100 µg/ml cycloheximide (+ CHX) and, after 30 min, CERKL levels in cell lysates were analyzed by Western blot with antibodies that recognize HA (upper panel) or, as a loading control, tubulin (lower panel). The numbers below indicate the relative amount of CERKL with respect to tubulin in each lane. (TIF) [file pone.0087898.s004.tif]

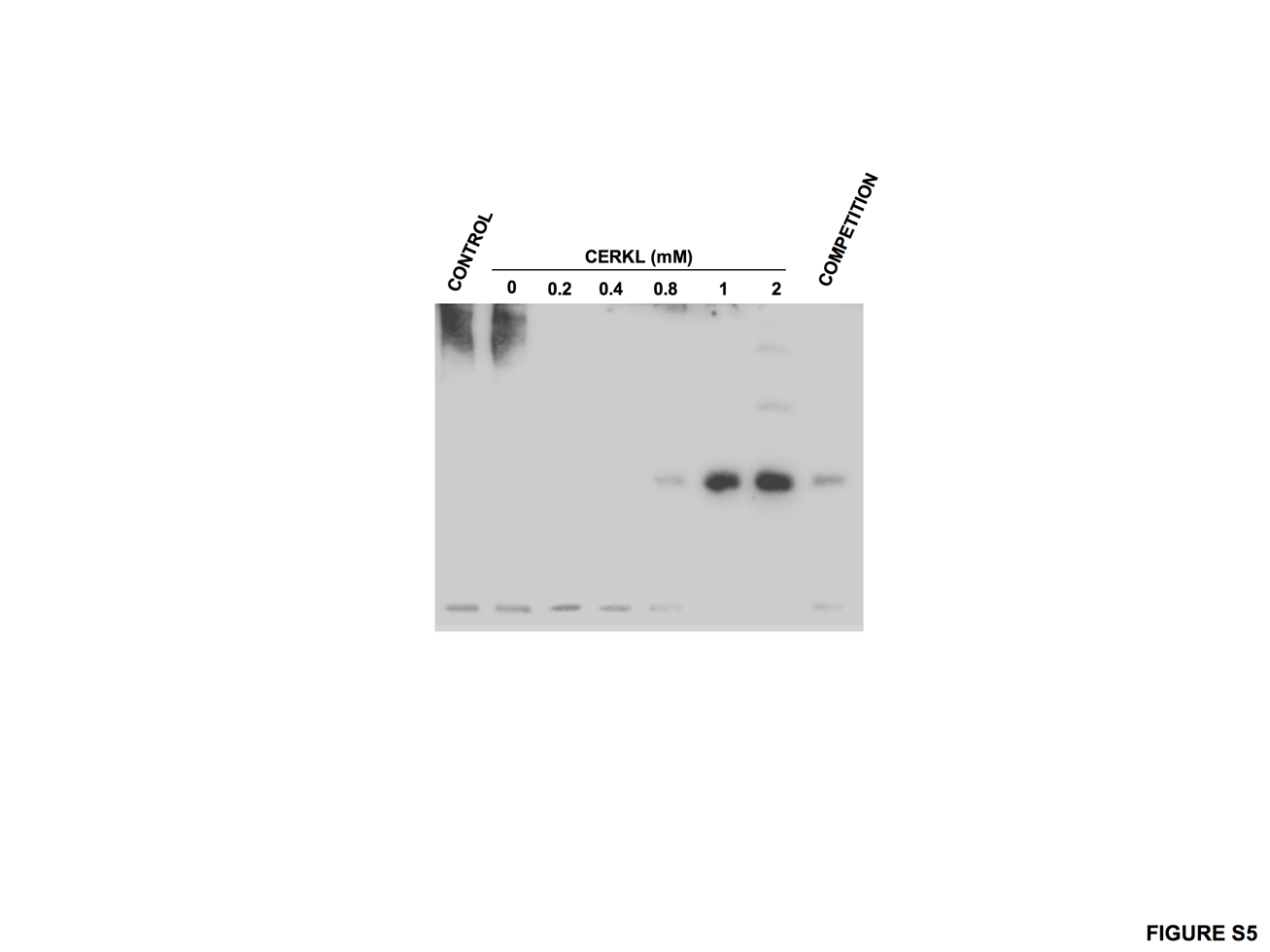

Supplement: Figure S5 — CERKL interacts with mRNAs. CERKL protein was purified and serial dilutions of the protein were incubated with biotinylated mRNAs from COS-7 cells. Specific shifted bands were observed (arrowheads) at protein concentrations as low as 0.8 µM in COS-7 mRNAs. Bovine serum albumin (first lane, CONTROL) and His-maltose binding protein (second lane, 0) were used as negative controls and addition of an excess of non-biotinylated probes reduced the intensity of the shifted bands (last lane, COMPETITION). (TIF) [file pone.0087898.s005.tif]

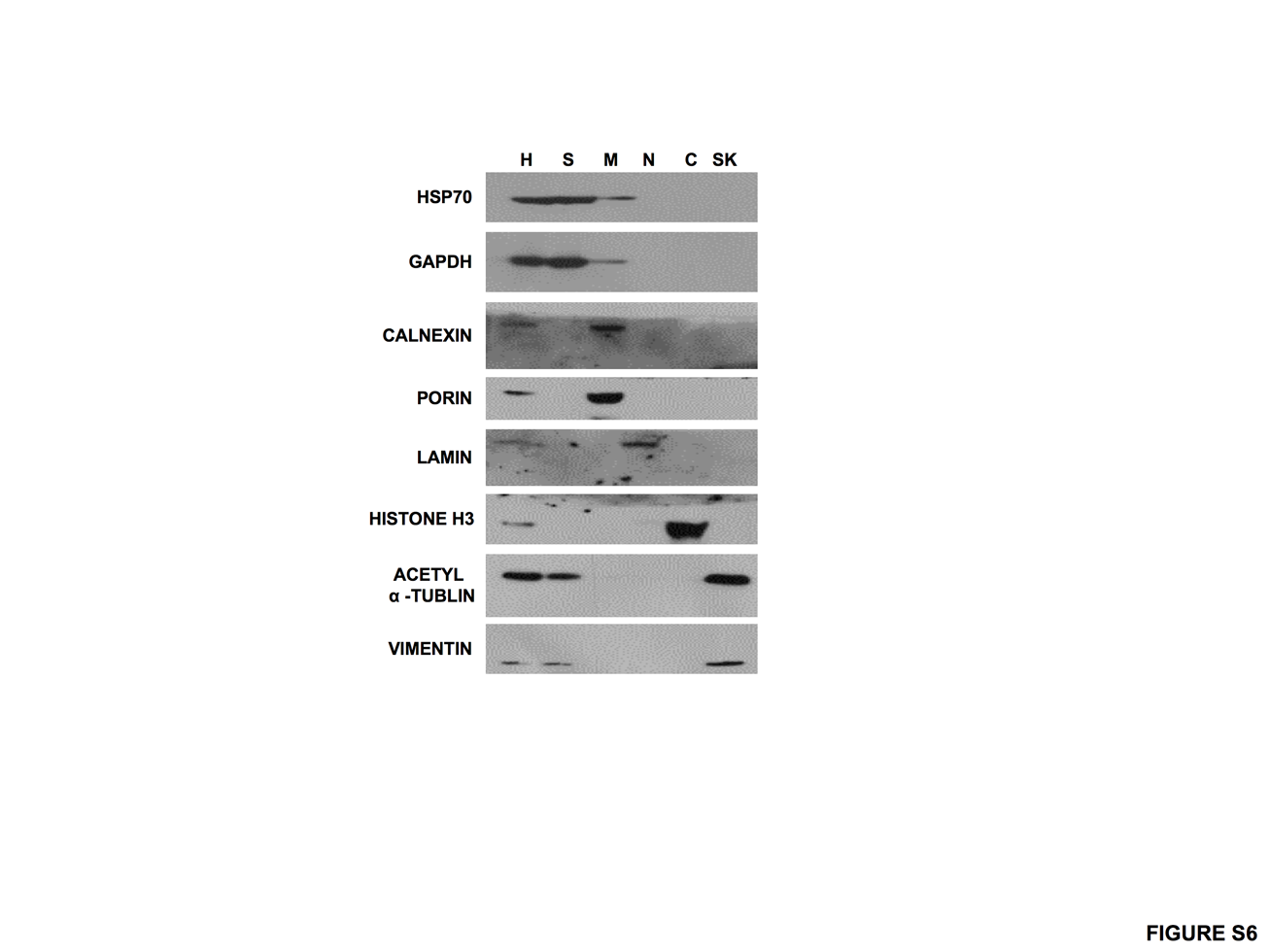

Supplement: Figure S6 — Isolation of a cytoskeletal fraction. Fractionation of HEK-293T cells to obtain a cytoskeletal fraction (SK) was carried out as described in Materials and Methods. Purity of each fraction was analyzed by Western blot using antibodies that recognize HSP70 and glyceraldehyde-3-phosphate dehydrogenase (GAPDH) as markers of the soluble fraction (S), calnexin and porin as markers of the membrane fraction (M), lamin and histone H3 as markers of nuclei (N) and chromatin (C), respectively, and acetyl-α-tubulin as a microtubule marker. Vimentin (intermediate filaments marker) was used as a control. H: total homogenate. (TIF) [file pone.0087898.s006.tif]
