# Supplementary material for: CERKL, a Retinal Disease Gene, Encodes an mRNA-Binding Protein That Localizes in Compact and Untranslated mRNPs Associated with Microtubules
Source: PLoS One. 2014 Feb 3;9(2):e87898. doi: 10.1371/journal.pone.0087898 (PMC3912138; doi:10.1371/journal.pone.0087898)
Supplement: Table S1 — CERKL interacting proteins. (DOC) [file pone.0087898.s007.doc]

**SUPPORTING TABLE S1. CERKL interacting proteins**.

| **Accession Number** | **Protein Name** | **Unused** | **Peptides (95%)** |
| --- | --- | --- | --- |
| **Proteins involved in translation and folding of proteins** | | | |
| sp|P55884|EIF3B_HUMAN | Eukaryotic translation initiation factor 3 subunit B | 22.83 | 11 |
| sp|O75821|EIF3G_HUMAN | Eukaryotic translation initiation factor 3 subunit G | 8.57 | 4 |
| sp|Q13347|EIF3I_HUMAN | Eukaryotic translation initiation factor 3 subunit I | 2.18 | 1 |
| sp|P26641|EF1G_HUMAN | Elongation factor 1-gamma | 4.92 | 3 |
| sp|P49411|EFTU_HUMAN | Elongation factor Tu, mitochondrial | 16.27 | 9 |
| sp|Q5VTE0|EF1A3_HUMAN | Putative elongation factor 1-alpha-like 3 | 12.71 | 8 |
| sp|Q05639|EF1A2_HUMAN | Elongation factor 1-alpha 2 | 2.75 | 2 |
| sp|P13639|EF2_HUMAN | Elongation factor 2 | 8.93 | 5 |
| sp|P49588|SYAC_HUMAN | Alanine-tRNA ligase | 8.78 | 6 |
| sp|P41252|SYIC_HUMAN | Isoleucine-tRNA ligase | 2.73 | 2 |
| sp|P56192|SYMC_HUMAN | Methionine-tRNA ligase | 4.22 | 3 |
| sp|P23396|RS3_HUMAN | 40S ribosomal protein S3 | 13.31 | 7 |
| sp|P08865|RSSA_HUMAN | 40S ribosomal protein SA | 4.22 | 2 |
| sp|P46782|RS5_HUMAN | 40S ribosomal protein S5 | 2.00 | 1 |
| sp|P08238|HS90B_HUMAN | Heat shock protein 90 | 36.93 | 19 |
| sp|P08107|HSP71_HUMAN | Heat shock protein 70 | 15.06 | 7 |
| sp|O60884|DNJA2_HUMAN | DNAJ homolog subfamily A member 2 | 6.51 | 4 |
| sp|P04792|HSPB1_HUMAN | Heat shock protein beta-1 | 4.03 | 2 |
| sp|P11940|PABP1_HUMAN | Poly A binding protein |  |  |
| **Proteins involved in DNA/RNA metabolism** | | | |
| sp|P06748|NPM_HUMAN  sp|Q9BQA1|MEP50_HUMAN  sp|Q9Y230|RUVB2_HUMAN  sp|P52597|HNRPF_HUMAN | Nucleophosmin  Methylosome protein 50  RuvB-like 2  Heterogeneous nuclear ribonucleoprotein F | 6.26  6.37  14.88  2.00 | 3  3  9  1 |
| **Others** | | | |
| sp|P05090|APOD_HUMAN  sp|Q8TEX9|IPO4_HUMAN  sp|Q9BQE3|TBA1C_HUMAN  sp|P07437|TBB5_HUMAN | Apolipoprotein D  Importin 4  α-tubulin  β-tubulin | 8.56  18.76  9.35  7.79 | 5  9  5  5 |

Proteins were immunoprecipitated with Flag-tagged CERKL or GFP and analyzed by liquid chromatography-tandem mass spectrometry as described in Materials and Methods. The twenty-seven identified proteins that interacted specifically with CERKL were classified into three categories. Accession numbers, unused values and the number of peptides identified with more than 95% of fidelity are included.

.
